# Comprehensive insights into the response of *Alexandrium tamarense* to algicidal component secreted by a marine bacterium

**DOI:** 10.3389/fmicb.2015.00007

**Published:** 2015-01-23

**Authors:** Xueqian Lei, Dong Li, Yi Li, Zhangran Chen, Yao Chen, Guanjing Cai, Xujun Yang, Wei Zheng, Tianling Zheng

**Affiliations:** ^1^State Key Laboratory of Marine Environmental Science and Key Laboratory of the Ministry of Education for Coastal and Wetland Ecosystems, School of Life Sciences, Xiamen UniversityXiamen, China; ^2^ShenZhen Research Institute of Xiamen UniversityShenZhen, China; ^3^Fujian Center for Disease Control and PreventionFuzhou, China

**Keywords:** *Alexandrium tamarense*, algicidal bacterium, photosynthetic responses, oxidative stress, antioxidant enzyme, gene expression inhibition

## Abstract

Harmful algal blooms occur throughout the world, threatening human health, and destroying marine ecosystems. *Alexandrium tamarense* is a globally distributed and notoriously toxic dinoflagellate that is responsible for most paralytic shellfish poisoning incidents. The culture supernatant of the marine algicidal bacterium BS02 showed potent algicidal effects on *A. tamarense* ATGD98-006. In this study, we investigated the effects of this supernatant on *A. tamarense* at physiological and biochemical levels to elucidate the mechanism involved in the inhibition of algal growth by the supernatant of the strain BS02. Reactive oxygen species (ROS) levels increased following exposure to the BS02 supernatant, indicating that the algal cells had suffered from oxidative damage. The levels of cellular pigments, including chlorophyll *a* and carotenoids, were significantly decreased, which indicated that the accumulation of ROS destroyed pigment synthesis. The decline of the maximum photochemical quantum yield (Fv/Fm) and relative electron transport rate (rETR) suggested that the photosynthesis systems of algal cells were attacked by the BS02 supernatant. To eliminate the ROS, the activities of antioxidant enzymes, including superoxide dismutase (SOD) and catalase (CAT), increased significantly within a short period of time. Real-time PCR revealed changes in the transcript abundances of two target photosynthesis-related genes (*psb*A and *psb*D) and two target respiration-related genes (*cob* and *cox*). The transcription of the respiration-related genes was significantly inhibited by the treatments, which indicated that the respiratory system was disturbed. Our results demonstrate that the BS02 supernatant can affect the photosynthesis process and might block the PS II electron transport chain, leading to the production of excessive ROS. The increased ROS can further destroy membrane integrity and pigments, ultimately inducing algal cell death.

## INTRODUCTION

Phytoplankton play an important role in marine ecosystems as it is the major primary producer in the euphotic layer (Thyssen et al., 2014). However, some of them cause harmful algal blooms (HABs), which are usually associated with severe damage to aquatic ecosystems, have been occurring more frequently worldwide (Anderson, 1997b; Siswanto et al., 2013). As a result, impacts on the environment, tourism, fisheries, and public health due to HABs, which disturb the balance of the marine ecosystem, have all increased over the past few decades (Anderson et al., 2012). *Alexandrium tamarense*, an important algal bloom-causing organism, is responsible for most incidents of paralytic shellfish poisoning in addition to damaging the aquatic environment and human health (Franks and Anderson, 1992; Anderson, 1997a).

To control HABs, both physical (Sengco and Anderson, 2004; Tang et al., 2011) and chemical approaches (Sun et al., 2004; Kim et al., 2012) have been carried out. However, such approaches are costly and have not succeeded in completely resolving the problems associated with HABs, and they have also been shown to have negative effects on the aquatic environment due to secondary pollution (Lee et al., 2001; Ni et al., 2012; Zheng et al., 2013). Therefore, biological control agents, including bacteria (Bai et al., 2011; Zhang et al., 2014), viruses (Cai et al., 2011; Sheik et al., 2013), macrophytes (Jin and Dong, 2003) and protozoa (Jeong et al., 2008), have gained increasing attention. In recent years, bacteria have been considered to play a crucial role in the dramatic termination of blooms in coastal seawaters (Paul and Pohnert, 2013). Most of the known algicidal bacteria have been assigned to the genera *Cellulophaga, Saprospira, Pseudoalteromonas*, *Alteromonas*, *Flavobacterium, Zobellia, Bacillus*, *Micrococcus*, *Planomicrobium*, *Pseudomonas,* and *Vibrio* (Mayali and Azam, 2004; Kim et al., 2009). These algicidal bacteria either directly or indirectly attack algal cells, the former requiring cell-to-cell contact (Mayali and Azam, 2004; Chen et al., 2014) and the latter producing substances with inhibitory effects on algal growth (Pokrzywinski et al., 2012).

Most reported algicidal bacteria have inhibitory effects on algal growth arising from the excretion of extracellular substances in a process termed allelopathy (Suikkanen et al., 2004). There are four aspects to be considered in relation to the potential pathways of the allelochemical inhibition of algal growth: the destruction of cell structures, alterations in enzymatic activities, and influences on algal photosynthesis or respiration (Peñuelas et al., 1996; Hong et al., 2009; Churro et al., 2010; Yang et al., 2011, 2013). During bloom succession, algal cells are subjected to diverse environmental stress conditions that lead to the production of reactive oxygen species (ROS), such as allelopathic interactions (Vardi et al., 2002), viral infection (Evans et al., 2006), UV exposure (Rijstenbil, 2002), CO_2_ availability (Vardi et al., 2007), and iron limitation (Thamatrakoln et al., 2012; Rosenwasser et al., 2014). Allelochemicals inhibit the photosynthetic efficiency and capacity of algal cells, causing them to produce numerous ROS (Apel and Hirt, 2004). To eliminate ROS and avoid oxidative damage, algal cells increase antioxidant defenses via the enzymatic and non-enzymatic antioxidants (Gill and Tuteja, 2010; Hamilton et al., 2012). In the enzymatic pathways, superoxide dismutase (SOD) and catalase (CAT) are important scavengers of ROS (Hong et al., 2009). However, when the production rate of ROS exceeds the scavenging rate, oxidative damage will occur, and many molecular sites will be attacked by the superfluous ROS (Avery, 2011).

Photosynthesis relies on the absorption of sunlight by chlorophyll molecules in photosystems I and II (PS I and II; Erickson et al., 1986). The *psb*A and *psb*D genes encode two core proteins, D1 and D2 of PS II (Marder et al., 1987). *cob* is a cytochrome *b*-related gene in the mitochondria, and *cox* is a cytochrome oxidase synthetic gene (Tobe et al., 2010). Previous studies have indicated that alterations occur in photosynthesis- and respiration-related gene expression in algae under allelochemical stress (Qian et al., 2010; Yang et al., 2013). However, few studies have focused on the damage caused by bacteria in relation to changes in photosynthesis- and respiration-related gene expression in *A. tamarense*.

To better understand the mechanism of the BS02 supernatant-mediated inhibition of the growth of *A. tamarense*, we assessed the effect of this supernatant at the physiological and biochemical levels by measuring ROS, the responses of antioxidant enzymes (SOD and CAT) to oxidative stress, and the transcript abundance of photosynthesis (*psb*A and *psb*D)- and respiration (*cob* and *cox*)-related genes.

## MATERIALS AND METHODS

### BACTERIAL AND ALGAL CULTURES

*Vibrio* sp. BS02 (GenBank No. HM596341.1) was isolated from a mangrove area in Zhangjiangkou, Fujian Province, China, and deposited into the Marin Culture Collection of China (MCCC) under accession number MCCC 1F01214. BS02 was cultured in marine agar 2216E broth (5 g peptone, 1 g yeast extraction, and 0.1 g ferric phosphorous acid, pH 7.4–7.8, brought to a total volume of 1 L using natural sea water), followed by incubation for 48 h at 28°C.

Cultures of the experimental alga, *A. tamarense* ATGD98-006, were supplied by the Algal Culture Collection, Institute of Hydrobiology, Jinan University, Guangzhou, China. The cultures were cultivated in f/2 medium prepared with natural seawater (Guillard, 1975) under a 12:12 h light–dark cycle with a light intensity of 50 μmol photons m^-2^s^-1^ at 20 ± 1°C.

### ANALYSIS OF ALGICIDAL MODE AND ACTIVITY

Strain BS02 was inoculated in 50 mL 2216E broth and grown to stationary phase at 28°C on a shaker at 150 rpm for 48 h. Then the cells were removed by centrifugation at 10,000 ×*g* for 10 min, and the supernatant was filtered through a 0.22 μm Millipore membrane. To assess whether the bacterial cells were completely removed from the filtrate, 150 μL aliquots of filtrate were spread onto marine agar 2216E plates followed by incubation for 3 days at 28°C. The remaining pellets were washed twice with sterile f/2 medium, and then the same volume of sterile f/2 medium was added to resuspend the cells.

Flasks (500 mL) were prepared, with each containing 100 mL of sterile f/2 algal culture medium. BS02 cell-free supernatant, washed bacterial cells and a bacterial culture prepared as described above were added to the axenic exponentially growing algal cultures at a proportion of 1.5% (v/v) in triplicate. Autoclaved 2216E broth served as a control. The algicidal rate was calculated according to the following formula (Li et al., 2014a):

(1) A⁢lgicidal⁢  activity(%)=(Nc−Nt)/Nc*100⁢

where N_C_ represents the number of algal cells in the control group, and N_t_ represents the number of algal cells in the treatment group.

Flasks (500 mL) were prepared, with each containing 100 mL of sterile f/2 algal culture medium. BS02 cell-free supernatant was added to the axenic exponentially growing algal cultures at proportions of 0.5 (v/v), 1.0 (v/v), and 1.5% (v/v) in triplicate. Autoclaved 2216E broth served as a control.

### SAMPLE PREPARATION AND SCANNING ELECTRON MICROSCOPY

The BS02 supernatant prepared as described above was added to axenic exponentially growing algal cultures at a concentration of 1.5% (v/v). Ten-milliliter aliquots of the culture medium were withdrawn based on algal cell vitality during the first day and then once every 12 h. The samples were fixed in 0.1 M sodium phosphate buffer (PBS, 8 g NaCl, 0.2 g KCl, 1.44 g Na_2_HPO_4_, 0.24 g KH_2_PO_4_, and 1 L distilled water; 50 mM, pH 7.4) containing 2.5% glutaraldehyde (v/v) for 2 h and then gently rinsed three times with PBS buffer followed by post-fixation in 1% OsO_4_ in the same buffer for 2 h. Next, the samples were gently rinsed three times with PBS buffer and dehydrated in a graded ethanol series (30, 50, 70, 90, 95, and 100%), and they were finally stored in pure tertiary butanol at 4°C overnight. The samples were subsequently critical-point-dried and mounted on stubs. The preparation was sputter coated with gold-palladium at 60:40 and 25–30 nm. The lysis process was visualized and imaged using scanning electron microscope (JSM-6390, JEOL).

### SAMPLE PREPARATION AND TRANSMISSION ELECTRON MICROSCOPY

Algal cells were treated with the BS02 supernatant at a proportion of 1.5% (v/v) for 48 h. Samples were collected by centrifugation at 2000 ×*g* for 15 min and then fixed overnight at 4°C in 0.1 M PBS buffer containing 2.5% glutaraldehyde (v/v). The fixed cells were rinsed three times in PBS buffer and post-fixed for 2 h in 1% OsO_4_ in the same buffer. After being washed three times, the samples were dehydrated in a graded ethanol series and then embedded in araldite resin. Sections (60–80 nm) obtained with an ultramicrotome were stained in 3% acetic acid uranium-citric acid and viewed using a JEM2100HC transmission electron microscope (JEOL Ltd., Tokyo, Japan).

### DETERMINATION OF ROS LEVELS

Intracellular ROS were detected following the protocol reported by Yin et al. (2005), with some modifications. The algal cells were incubated with 2′, 7′- dichlorofluorescin diacetate at 37°C in the dark for 1 h at a final concentration of 10 μM and mixed every 5 min during this time. Then, following three washes with sterile f/2 medium, the cells were resuspended in 1 mL of this medium. Fluorescence intensity was monitored at an excitation wavelength of 488 nm and an emission wavelength of 525 nm using a spectrofluorometer.

### LIPID PEROXIDATION AND ANTIOXIDATIVE ENZYME ASSAYS OF *A. tamarense*

Algal cells were collected by centrifugation at 5000 ×*g* for 5 min, and the pellets were washed twice with sterile seawater. The cells were then resuspended in 1 mL PBS solution (50 mM, pH 7.4), and cell disruption was conducted using an Ultrasonic Cell Disruption System (NingBo Scientiz Biotechnological Co., Ltd., China; 80 W, ultrasonic time of 5 s and rest time of 5 s for 40 cycles) at temperatures of below 4°C. Cell debris was removed by centrifugation at 10,000 ×*g* for 10 min at 4°C. The supernatant was used to analyze the level of malondialdehyde (MDA) and the activities of SOD and CAT. All analysis methods were preformed according to the manufacturers’ instructions at the Nanjing Jiancheng Bioengineering Institute, China (Zhang et al., 2011).

### PIGMENTS AND CHLOROPHYLL FLUORESCENCE MEASUREMENTS

The chloroplast pigment contents were measured based on previously described methods (Inskeep and Bloom, 1985) with slight modifications. Ten milliliters of algal culture was collected by centrifugation at 5000 ×*g* for 10 min and washed with PBS (50 mM, pH 7.4). Then, the pellets were resuspended in 90% ethanol overnight at 4°C to extract the chloroplast pigments. After complete extraction, the pellets were removed by centrifugation at 4°C for 10 min at 8000 ×*g*, and the supernatant was used to measure absorbance at wavelengths of 665, 645, and 470 nm. The pigments were calculated as follows:

(2) $$Chlorophyll\,\;\;a(mg/L)=12.7*A_{665}-2.69*A_{645}$$

(3) $$Carotinoid\,\;\;(mg/L)=\left(1000*A_{470}-2.05*C_{Chlorophyll\,\;\;\;\;\;a}\right)/245\,$$

where A_665_, A_645,_ and A_470_ represent absorbance values at wavelengths of 665, 645, and 470 nm, respectively, and C_Chlorophyll_ *_a_* indicates the content of chlorophyll *a* (Chl *a*).

Pulse-amplitude modulation fluorescence measurements were obtained using a PAM-CONTROL Fluorometer (Walz, Effeltrich, Germany). The maximum photochemical quantum yield (Fv/Fm) were determined under an actinic light of 3000 μmol photons m^-2^ s^-1^ based on previously described methods (Drábková et al., 2007) after the algal cells were dark-adapted for 15 min. The relative electron transport rate (rETR/μmol electrons m^-2^ s^-1^) were measured followed (Kaplan et al., 2013), eight consecutive light levels of 156, 226, 337, 533, 781, 1077, 1593, and 2130 μmol photons m^-2^ s^-1^ were applied at 15 s intervals.

### RNA EXTRACTION AND QUANTITATIVE REAL-TIME PCR ANALYSIS

Fifty milliliters of algal culture was treated with the BS02 supernatant for 4 h and 24 h and then collected at 3000 × g for 5 min at 4°C, and the pellets were quickly frozen in liquid nitrogen. Total RNA was extracted as quickly as possible using an RNAiso Kit (TaKaRa Company, China) following the manufacturer’s instructions. Reverse transcription of the RNA was performed using a PrimeScript^TM^ RT-PCR Kit (TaKaRa Company, China). Two important photosynthesis genes (*psb*A and *psb*D) and two target respiration-related genes (*cob* and *cox*) were selected for real-time qPCR analysis. Real-time PCR was carried out using SYBR^®^ Premix EX Taq^TM^ II (TaKaRa Company, China). The 18S rRNA gene was used as a reference gene to standardize the results. All of the primer pairs are listed in **Table 1**. The RT-PCR program was as follows: denaturation at 95°C for 20 s and 45 cycles of 95°C for 10 s followed by 60°C for 20 s and 72°C for 20 s. The relative gene expression among the treatment groups was quantified using the 2^-ΔΔCt^ method (Livak and Schmittgen, 2001).

**Table 1:** Sequences of primer pairs used with *Alexandrium tamarense* for real-time PCR.

Gene name	Sequence (5′–3′)
18S rRNA	F:5′-GAATTCCTAGATATCGCAGTTCATC-3′ R:5′-GCTAATCCACAATCTCGACTCCTC-3′
*psb*A	F: 5′-CAATGACAGTACGCCACCAA-3′ R: 5′-CGAGATCAGCCTAGCTATTT-3′
*psb*D	F: 5′-TCTGTTACTTTACTTGATGACTGG-3′ R: 5′-AGACAATGAAATGAACTACTGACC-3′
*cob*	F:5′-AGCATTTATGGGTTATGTNTTACCTTT-3′ R:5′-AGCTTCTANDGMATTATCTGGATG-3
*cox*	F: 5′-GAGGTGGAACAGGATGGA-3′ R: 5′-GTGTAACAATGGCGGATT-3′

## RESULTS

### ALGICIDAL MODE AND ACTIVITY OF BS02 SUPERNATANT

**FIGURE 1:**
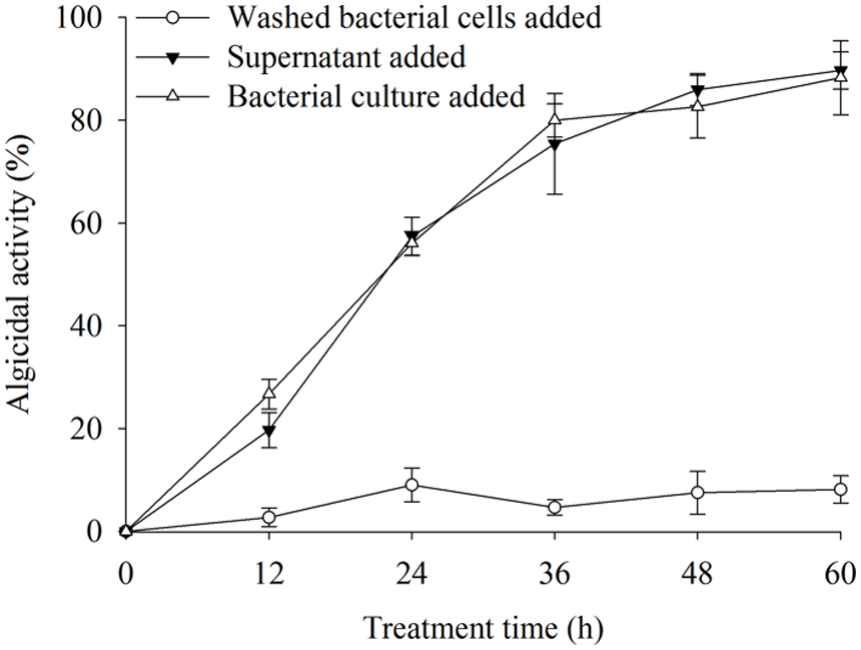
**The algicidal activity of different parts of bacterial cultures.** All error bars indicate SE of the three replicates.

The algicidal activities were 88.9 and 89.6% after 60 h of treatment with the BS02 cell-free supernatant and bacterial cultures (**Figure 1**). We found that the cell-free supernatant showed high algicidal activity, which was similar to that of the bacterial cultures, whereas the washed bacterial cells did not show any obvious algicidal activity.

**FIGURE 2:**
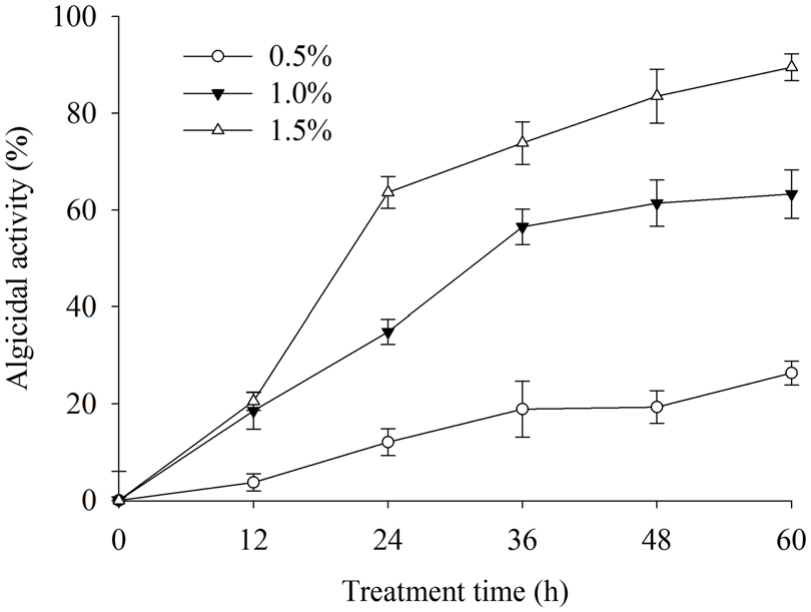
**Effect of different volume of BS02 supernatant on *Alexandrium tamarense.*** All error bars indicate SE of the three replicates.

To determine the effective algal-lytic concentration of BS02 against *A. tamarense,* different proportions of the BS02 supernatant (0.5, 1.0, and 1.5%) were inoculated in algal cultures. The proportion of 0.5% supernatant showed lower algal-lytic activity compared with the other two groups. The algicidal activities were 26.3, 63.2, and 89.5% after 60 h of treatment with the BS02 supernatant at the 0.5, 1.0, and 1.5% concentrations (**Figure 2**).

### EFFECTS OF BS02 SUPERNATANT ON MORPHOLOGICAL AND SUBCELLULAR STRUCTURES

**FIGURE 3:**
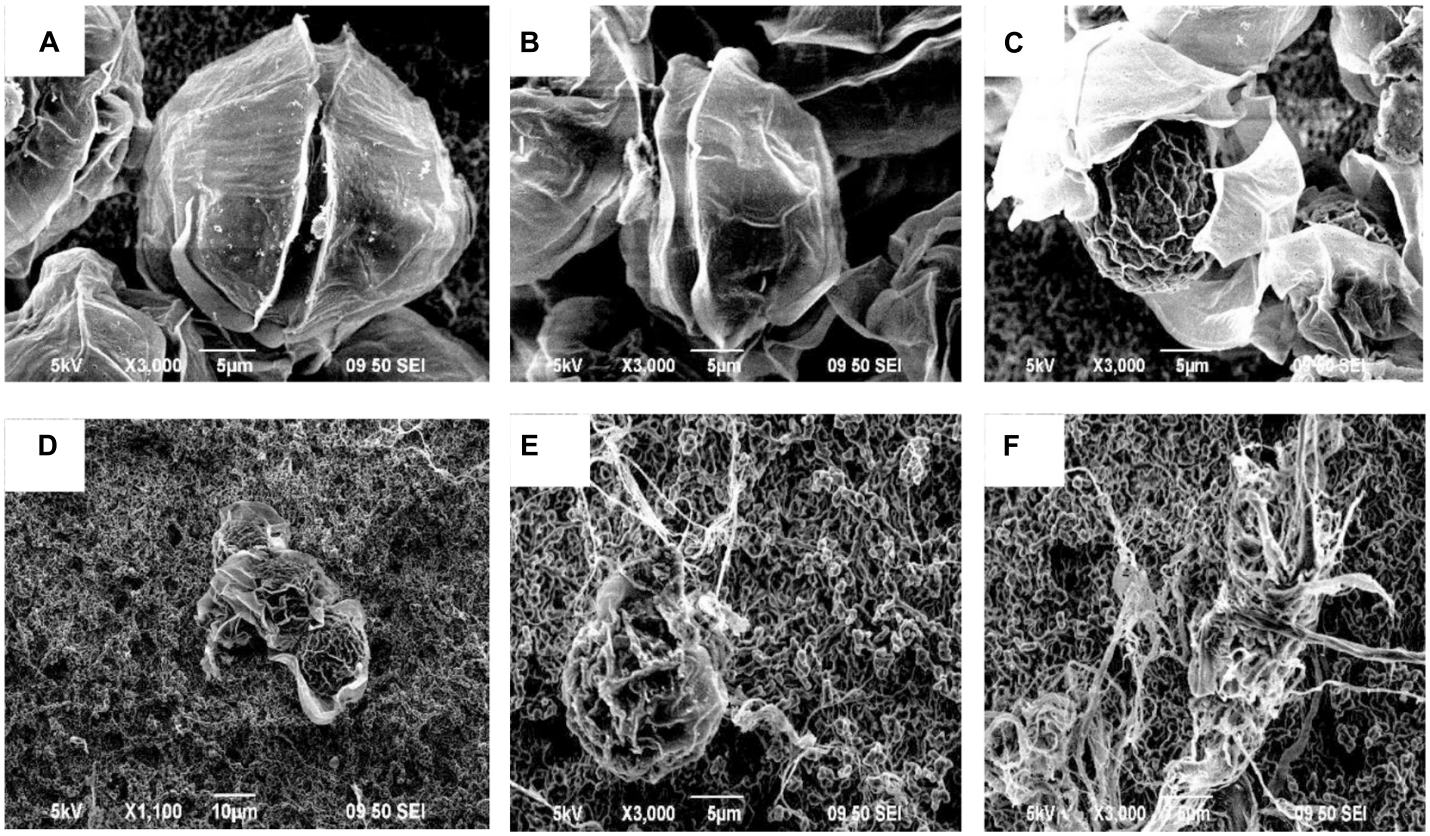
**Morphological changes of *A. tamarense* cells treated with BS02 supernatant at a concentration of 1.5% under a scanning electron microscope.** Bars, **(A–C,E,F)** 5 μm; **(D)** 10 μm.

Based on the SEM observations and optical observations of samples from several time points, the algicidal process was inferred. **Figure 3** shows the morphological changes of the *A. tamarense* cells, which were treated with the BS02 supernatant at a concentration of 1.5%. **Figure 3A** shows the normal cells in the control, which exhibited integrity of the cell wall and cell membrane, a visible fissure in the middle of the cell wall, and a quasi-spherical shape. Compared with these normal cells, the treated cells showed many differences in morphological characteristics and even structural damage (**Figures 3B–F**). The cells gradually became wrinkled, and one side of the cell wall began to appear sunken after 12 h of treatment (**Figure 3B**). After 24 h of treatment, the cells gradually shrank and the cytoplasm showed marked separation (**Figure 3C**). With increased exposure time, disintegration of the cell wall could be observed, the *A. tamarense* cells lysed, and part of the cellular substances were decomposed and released from the cell (**Figures 3D,E**). After 60 h of treatment, the *A. tamarense* cells were completely lysed and the cellular substances were completely decomposed and released (**Figure 3F**).

**FIGURE 4:**
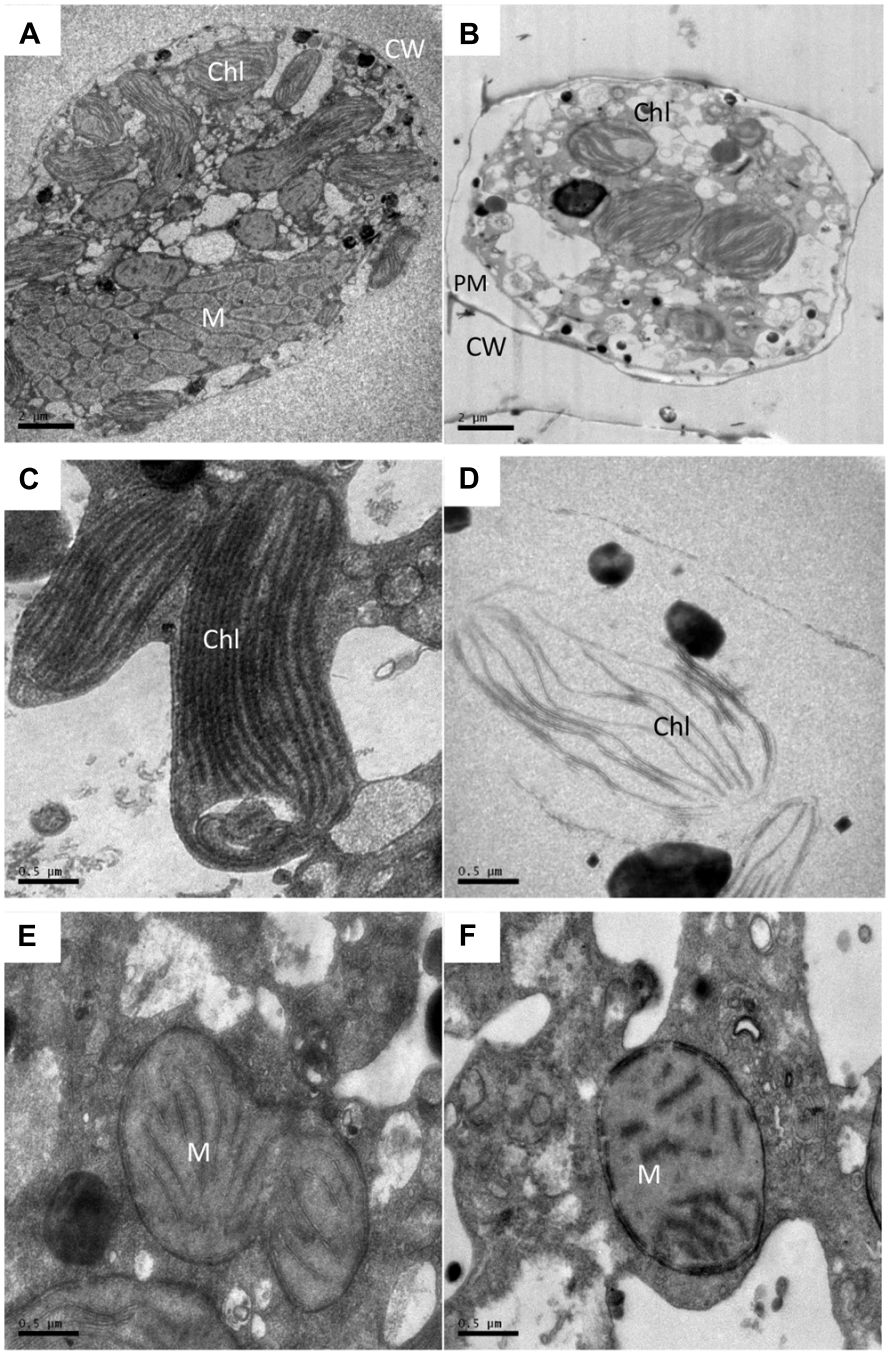
**Ultrastructure of *A. tamarense* after exposure to BS02 supernatant with a concentration of 1.5% for 48 h. (A,C,E)**: Control cell; **(B,D,F)**: treatment cells. Abbreviations: CW, cell wall; Chl, chloroplast; M, mitochondrion; PM, plasma membrane; Bars **(A,B)** 2 μm; **(C–F)** 0.5 μm.

Transmission electron microscopy analysis showed apparent alterations in the ultrastructure of *A. tamarense* due to the effects of the BS02 supernatant. As shown in **Figure 4**, the structure and morphology of the algal cells were altered, and obvious plasmolysis and vacuolization could be observed following exposure to the BS02 supernatant (**Figure 4B**). In the control group, both the chloroplasts and mitochondria were intact and possessed double membranes (**Figures 4C,E**). Obvious modifications appeared in the chloroplasts and mitochondria of the treated algal cells; for example, the chloroplasts became sparse and their membrane structures were severely damaged (**Figure 4D**). In addition, the cristae of the mitochondria were distorted and their membranes were partially disintegrated (**Figure 4F**).

### EFFECTS OF REACTIVE OXYGEN SPECIES AND MDA LEVELS

**FIGURE 5:**
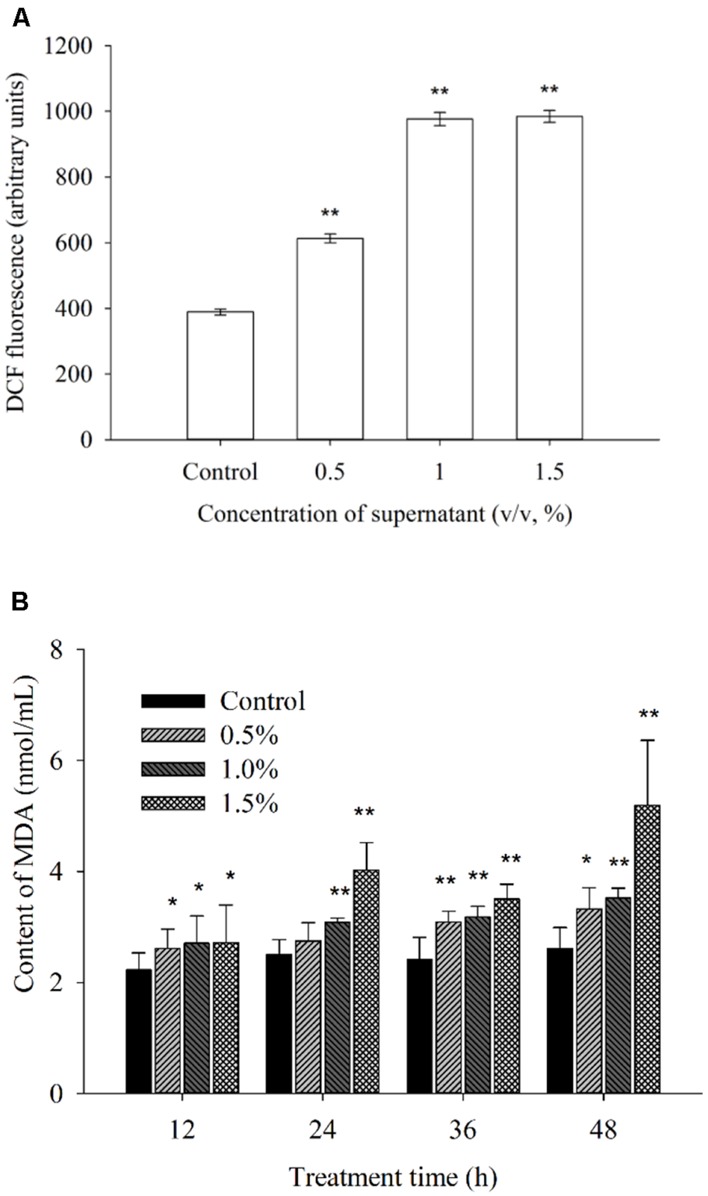
**Effects of BS02 supernatant on the ROS **(A)** and MDA **(B)** contents of *A. tamarense*.** All error bars indicate SE of the three replicates.*represents a statistically significant difference of *p* < 0.05 when compared to the control, **represents a statistically significant difference of *p* < 0.01.

To investigate whether exposure to different supernatant concentrations of BS02 damages algal cells, ROS levels and membrane lipid peroxidation were measured. ROS and lipid peroxidation levels, which are two important parameters, indicated the oxidative damage of the cellular components. **Figure 5A** shows the fluorescence intensity of ROS after 2 h of treatment with the supernatant. The ROS level was significantly (*p* < 0.01) increased in the treatment group compared with the control group. Intracellular ROS levels after 2 h of treatment with concentrations of 0.5, 1.0, and 1.5% of the supernatant were 1.58 (*p* < 0.01), 2.51 (*p* < 0.01), and 2.53 times (*p* < 0.01) those of the control, respectively.

Malondialdehyde can reflect the degree of lipid peroxidation, and it is used for evaluating the degree of cell damage. **Figure 5B** shows the MDA levels. After 12 h of treatment, the MDA level increased significantly (*p* < 0.05) compared to the control (**Figure 5B**). As the treatment time was prolonged, the MDA level was higher than that of the control group at each concentration. The MDA levels after treatment with the 1.5% concentration for 12, 24, 36, and 48 h were 1.21 (*p* < 0.05), 1.60 (*p* < 0.01), 1.45 (*p* < 0.01), and 1.99 times (*p* < 0.01) those of the control, respectively. Within 48 h of treatment, these levels were approximately 1.27 (*p* < 0.05), 1.35 (*p* < 0.01), and 1.99 times (*p* < 0.01) those of the control after exposure to the 0.5, 1.0, and 1.5% concentrations of the supernatant, respectively.

### EFFECTS OF ANTIOXIDATIVE ENZYME ACTIVITIES

**FIGURE 6:**
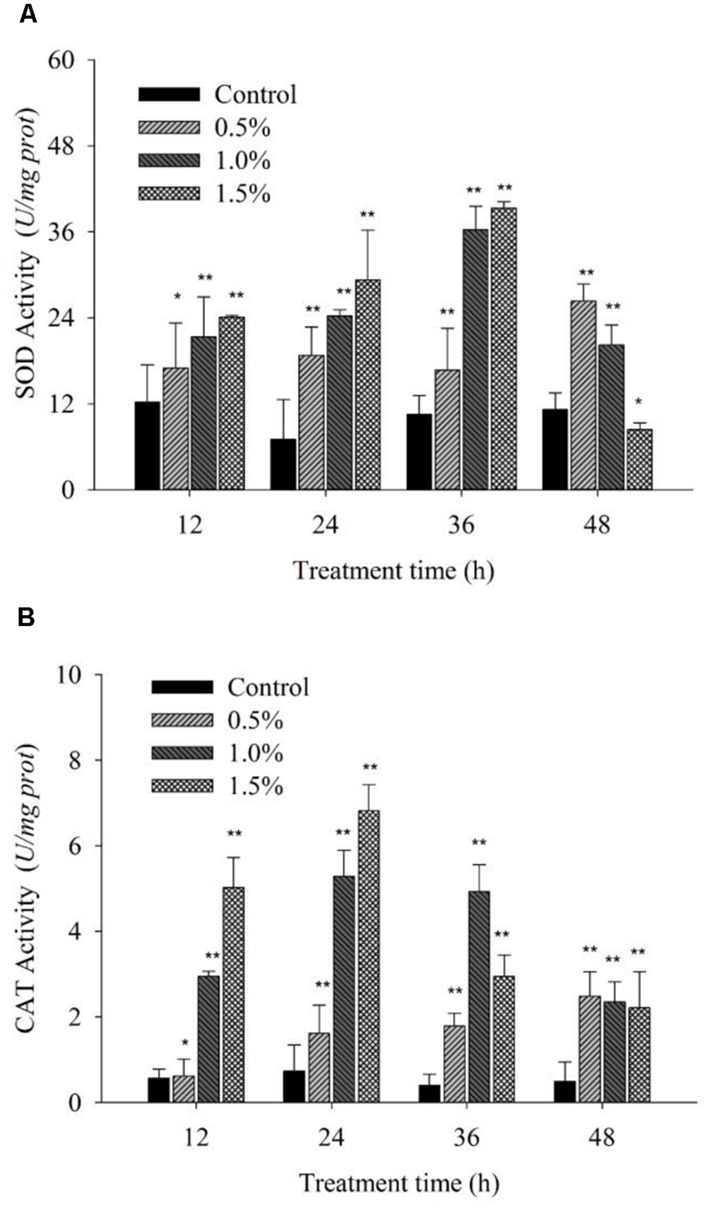
**Effects of BS02 supernatant on the SOD **(A)** and CAT **(B)** contents of *A. tamarense*.** All error bars indicate SE of the three replicates.*represents a statistically significant difference of *p* < 0.05 when compared to the control, **represents a statistically significant difference of *p* < 0.01.

Cellular enzymatic activities, including those of SOD and CAT, were measured to investigate the cellular defense response induced by the BS02 supernatant. **Figure 6A** shows that SOD activity increased significantly (*p* < 0.05) compared with the control after the algal cells were treated for 12 h. The activity values were 1.39 (*p* < 0.05), 1.75 (*p* < 0.01) and 1.97 times (*p* < 0.01) those of the control when the algal cells were treated with 0.5, 1.0, and 1.5% of the BS02 supernatant, respectively. SOD activity increased gradually as the treatment time was prolonged, increasing to maximum levels after 36 h of treatment to values of 1.58 (*p* < 0.01), 3.45 (*p* < 0.01), and 3.74 times (*p* < 0.01) those of the control after exposure to the supernatant at concentrations of 0.5, 1.0, and 1.5%, respectively. However, SOD activity decreased significantly after the 36 h treatment at the 1.0 and 1.5% concentrations, showing that longer exposure times did not induce a significant increase in the activity of this enzyme.

**Figure 6B** shows that CAT activity increased significantly (*p* < 0.01) at the 1.0 and 1.5% concentrations compared with the control during the first 12 h of exposure, and its activity continued to increase to a maximal level over 24 h. However, as the treatment time was prolonged, CAT activity decreased, although it was still obviously higher compared with the control, which is similar to the results for SOD. Although CAT activity decreased following treatment with the 1.0 and 1.5% concentrations as the exposure time was prolonged, it was also significantly (*p* < 0.01) higher compared with the control. The CAT activity values were 2.21 (*p* < 0.01), 7.22 (*p* < 0.01), and 9.31 times (*p* < 0.01) those of the control after exposure to the 0.5, 1.0, and 1.5% concentrations of supernatant for 24 h, respectively.

### PIGMENT CONTENTS AND PHOTOSYNTHESIS EFFICIENCY ANALYSIS

**FIGURE 7:**
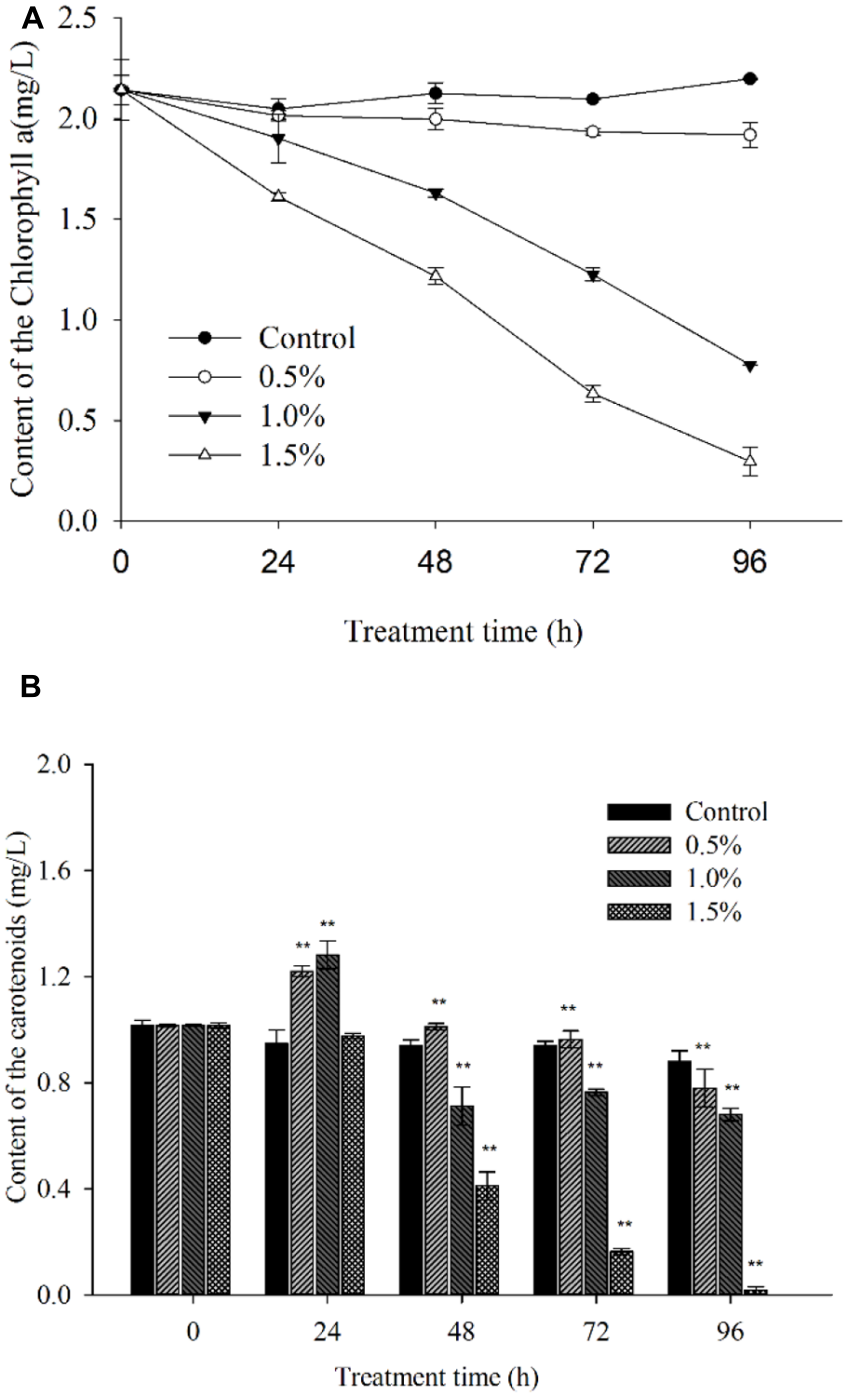
**Inhibitory effects of BS02 supernatant on chlorophyll *a***(A)** and carotenoid **(B)** content in *A. tamarense*.** All error bars indicate SE of the three replicates. **represents a statistically significant difference of *p* < 0.01 when compared to the control.

In the process of algal growth, the cellular pigments (Chl *a* and carotenoid) in the algal cells were destroyed due to the effects of the supernatant at concentrations of 1.0 and 1.5% (**Figure 7**) compared with the control and 0.5% concentration. At 96 h of exposure, its inhibitory effects reached the highest level, and the levels of Chl *a* were ∼35.3 and 13.5% of those of the control after exposure to the supernatant at concentrations of 1.0 and 1.5%, respectively (**Figure 7A**). However, the Chl *a* level showed no obvious change at the 0.5% concentration compared with the control.

**Figure 7B** indicates that the carotenoid concentration increased significantly compared with the control within 24 h of exposure and decreased after 24 h of exposure at the 0.5 and 1.0% supernatant concentrations. Following 48 h of treatment, the carotenoid level was significantly (*p* < 0.01) decreased due to the effects of the supernatant at concentrations of 1.0 and 1.5%, and its levels were approximately 75.7 and 43.8% of the control after exposure to the 1.0 and 1.5% concentrations, respectively. After 96 h of exposure, the carotenoid levels were significantly (*p* < 0.01) decreased at the supernatant concentration of 1.5%, and its levels were found to be 88.6, 77.3, and 20.4% of the control level after exposure to the 0.5, 1.0, and 1.5% concentrations of supernatant, respectively.

**FIGURE 8:**
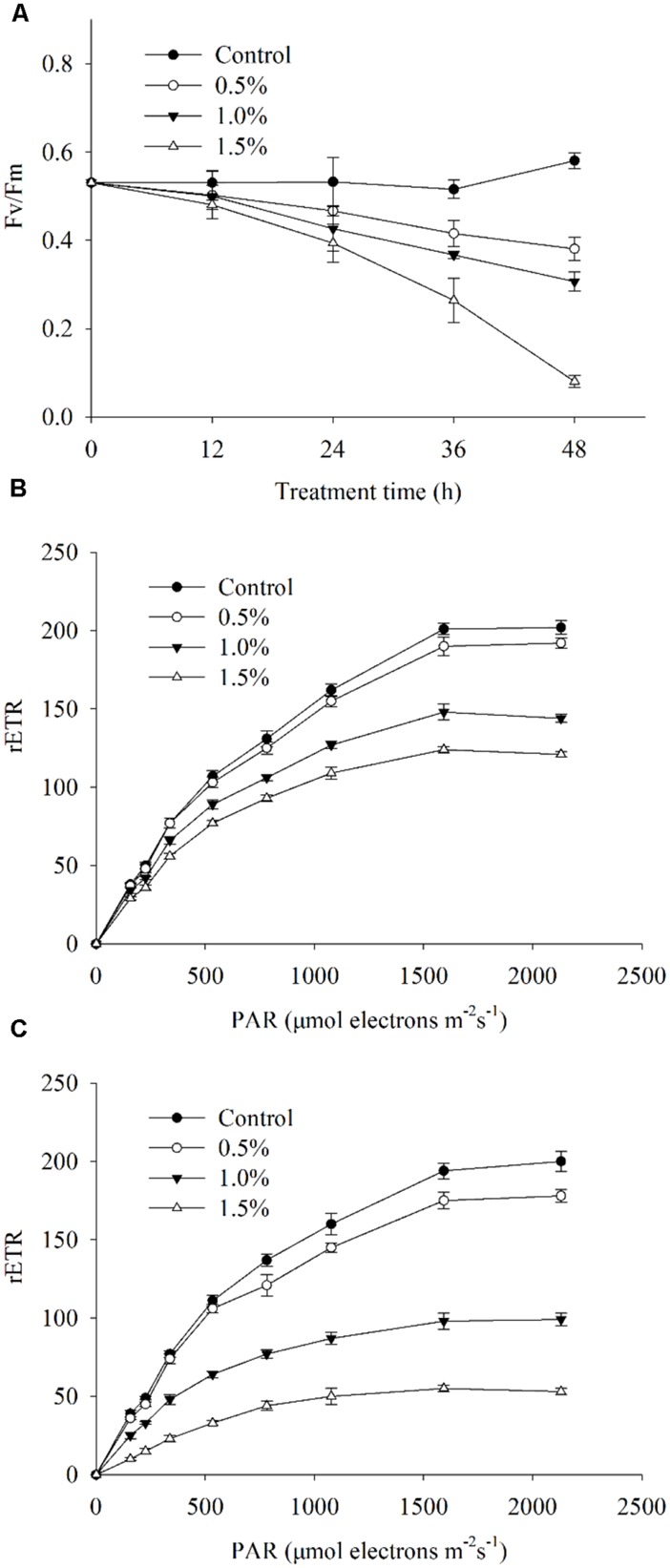
**Photosynthetic efficiency (Fv/Fm) **(A)**, photosynthetic capacity (rETR) exposed for 4 h **(B)** and rETR exposed for 24 h **(C)** of *A. tamarense* cells treated with different concentrations of BS02 supernatant.** All error bars indicate SE of the three replicates.

To investigate the photosynthetic status of the algal cells under the stress caused by the BS02 supernatant, we calculated the Fv/Fm and the rETR values, which, respectively, represented the photosynthetic efficiency and capacity of the cells. Within 1 h of treatment, the treatment group values were slightly lower than those of the control. The Fv/Fm values showed significant decreases (*p* < 0.01) after 12 h of exposure to the BS02 supernatant (**Figure 8A**), and after 48 h of exposure, the values were approximately 65.6, 52.8, and 13.8% of those of the control after exposure to the supernatant at concentrations of 0.5, 1.0, and 1.5%, respectively, implying that the inhibition of Fv/Fm was caused by the supernatant. Overall, the 1.5% treatment group showed a lower Fv/Fm value compared with the other treatments and the control (*p* < 0.01). The rETR, which was used to evaluate the photosynthetic capacity, showed a similar results with Fv/Fm (**Figures 8B,C**). **Figure 8B** shows that rETR values decreased significantly (*p* < 0.01) at the 1.0 and 1.5% concentrations compared with the control during the first 4 h of exposure. Within the 24 h treatment, the rETR values in the 1.0 and 1.5% treatment groups decreased obviously (*p* < 0.01) compared with the 4 h treatment groups (**Figure 8C**). However, the rETR values of the 0.5% treatment group were slightly lower than those of the control group after treatment for 4 and 24 h (**Figures 8B,C**).

### EXPRESSION OF PHOTOSYNTHESIS-RELATED GENES AND RESPIRATION-RELATED GENES

**FIGURE 9:**
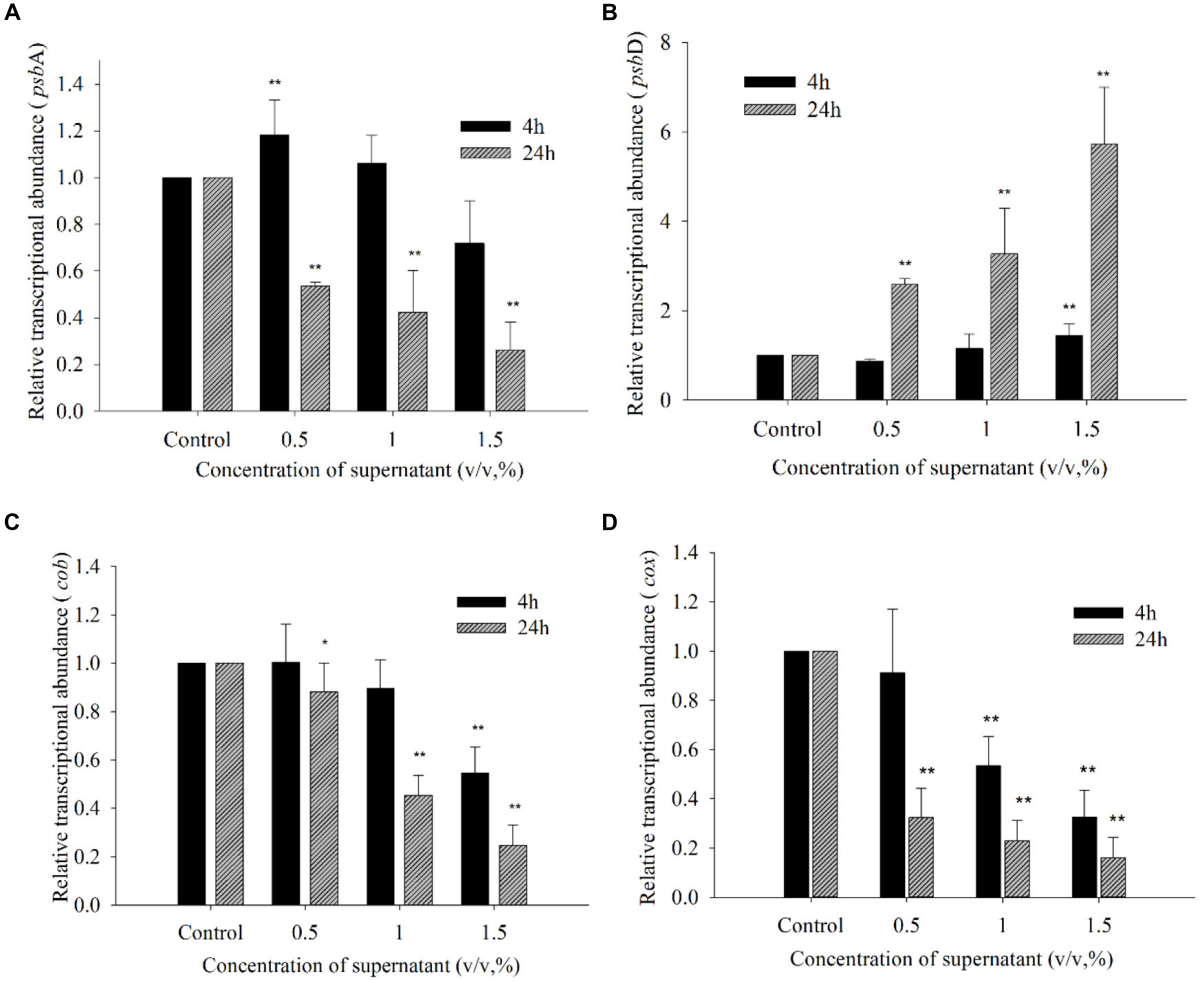
**Expression of *psb*A **(A)**, *psb*D **(B)**, *cob***(C),** and *cox***(D)** in *A. tamarense* exposed to different concentrations of BS02 supernatant supernatant for 4 and 24 h.** All error bars indicate SE of the three replicates.*represents a statistically significant difference of *p* < 0.05 when compared to the control, **represents a statistically significant difference of *p* < 0.01.

**Figure 9** shows the effects of the BS02 supernatant on the relative transcript abundances of photosynthesis- and respiration-related genes after 4 and 24 h of exposure. To identify whether *psb*A responded to the BS02 supernatant, its expression was analyzed using qRT-PCR in cells that had been treated with BS02 supernatant for 4 and 24 h. *psb*A transcript abundance was significantly affected by exposure to the BS02 supernatant (**Figure 9A**). Within 24 h of exposure, *psb*A gene expression in all treatment groups was significantly inhibited (*p* < 0.01) compared to the control, and the abundance of its transcript was 0.54, 0.42, and 0.26 times that of the control at the 0.5, 1.0, and 1.5% concentrations, respectively. However, transcription was stimulated during the initial exposure stage (4 h) in the 0.5% (*p* < 0.01) group compared to the control. The relative expression of *psb*A was 1.18 times that of the control at the 0.5% concentration after 4 h of exposure.

The transcription of *psb*D exhibited a somewhat different response to the BS02 supernatant compared with *psb*A (**Figure 9B**). Within 4 h of exposure to the 0.5% concentration, the transcription of *psb*D decreased to 0.86 times that of the control. However, this gene was up-regulated at all concentrations compared with the control after 24 h of exposure, and its expression levels were 2.58 (*p* < 0.01), 3.27 (*p* < 0.01), and 5.72 times (*p* < 0.01) those of the control in the 0.5, 1.0, and 1.5% treatment groups, respectively. Thus, *psb*D transcription was increased at the higher treatment concentrations, especially the 1.5% concentration.

The transcription of *cob* was inhibited by the supernatant at concentrations of 1.0 and 1.5% after 4 h of exposure (**Figure 9C**). Within 24 h of exposure, the relative expression levels of *cob* were 0.88 (*p* < 0.05), 0.45 (*p* < 0.01), and 0.25 times (*p* < 0.01) those of the control at the 0.5, 1.0, and 1.5% concentrations, respectively. The abundance of the *cox* transcript was significantly inhibited by the BS02 supernatant (**Figure 9D**). Within 4 h of exposure, *cox* transcription was inhibited by 0.91- (*p* < 0.01), 0.53- (*p* < 0.01), and 0.33-fold (*p* < 0.01) compared with the control in the 0.5, 1.0, and 1.5% treatment groups, respectively. The relative expression levels of *cox* were 0.26 (*p* < 0.01), 0.12 (*p* < 0.01), and 0.10 times (*p* < 0.01) those of the control at the 0.5, 1.0, and 1.5% concentrations after 24 h of exposure, respectively.

## DISCUSSION

### ALGICIDAL MODE OF BS02 SUPERNATANT AND LYSIS PROCESS OF *A. tamarense*

Blooms of *A. tamarense* species are associated with the largest number of paralytic shellfish poisoning cases around the world. There is an urgent need to seek effective methods to control the development of these blooms and to explore the associated mechanism of algal lysis. To date, there are few reports on the effects of bacteria on *A. tamarense*. To further explore the mechanism of the effects of the BS02 supernatant on this dinoflagellate, morphological changes, ROS levels, lipid peroxidation products, enzymatic antioxidants, pigment contents, Fv/Fm, and gene expression were assessed.

Algicidal bacteria can directly or indirectly attack algal cells, the former requiring cell-to-cell contact (Mayali and Azam, 2004) and the latter depending on active compounds produced by the microorganism (Guan et al., 2014). The results of this study revealed that the strain BS02 lysed *A. tamarense* cells via active algicidal compounds in the cell-free supernatant (**Figure 1**). We found that the cell-free supernatant had high algicidal activity, similar to that of the bacterial cultures. However, negligible algicidal activity was shown for the washed bacterial cell treatment. These results implied that the algicidal mode of strain BS02 was indirect attack via the release of algicidal compounds rather than direct contact with the algal cells.

Scanning electron microscope and TEM were used in this study to visualize the lysis process and to reveal alterations in the ultrastructure of *A. tamarense*. We observed obvious morphological (**Figure 3**) and ultrastructure changes (**Figure 4**) of the *A. tamarense* cells induced by the BS02 supernatant compared to the normal cells. Obvious modifications appeared in the chloroplasts and mitochondria of the treated algal cells. Ultrastructural changes in chloroplasts can inhibit photosynthetic activity by decreasing electron transport, photon absorption and the reaction center of PS II in algae (Li et al., 2014b). TEM analysis also showed that the BS02 supernatant damaged the structure of the mitochondria, which disrupted normal respiration and induced algal death.

### EFFECT OF ROS LEVELS, MDA LEVEL AND ANTIOXIDATIVE ENZYME ACTIVITY

Reactive oxygen species, including hydrogen peroxide, superoxide anion radicals and hydroxyl radicals, are unavoidable byproducts of oxygenic photosynthesis that cause oxidative damage and, ultimately, cell death (Alboresi et al., 2011; Yang et al., 2011). The inhibition of photosynthesis induces cells to produce numerous ROS, and excessive ROS cause lipid peroxidation and lead to severe cellular injury or death (Apel and Hirt, 2004; Bayr, 2005). The chloroplasts of algal cells, which exhibit intense electron flow, can produce ROS at high rates, causing oxidative damage to algal cells (Pérez-Pérez et al., 2012). Indirect damage by ROS includes the inhibition of photosynthesis, lipid peroxidation and photosynthetic oxidation (He and Häder, 2002). Our results demonstrated that the BS02 supernatant could significantly increase ROS levels in algal cells after 2 h of exposure (**Figure 5A**). At the 0.5 and 1.0% supernatant concentrations, ROS levels were significantly (*p* < 0.01) higher than those of the control, which indicated that the BS02 supernatant increased intracellular ROS significantly within a short period of time. At the highest treatment concentration, the ROS levels were markedly similar to those observed at the 1.0% concentration, indicating that excessive ROS were cleared by the antioxidative system of the algal cells. To characterize the lipid peroxidation level caused by ROS, the MDA level was measured.

Malondialdehyde is an indicator of lipid peroxidation and can reflect cellular oxidative damage, and Draper and Hadley (1990) have reported its use as an index of lipid peroxidation. In our study, the MDA level increased along with the BS02 supernatant concentration and duration of exposure. As shown in **Figure 5B**, we observed that the MDA level in all treatment groups increased compared to that in the control (*p* < 0.05) within 12 h of exposure. As the treatment time was prolonged, the MDA level was higher than that in the control group for each concentration. The 1.5% supernatant-treated group showed a higher level of MDA than the groups treated with lower concentrations, implying that the higher the concentration, the more serious the oxidative damage to algal cells. These results showed that increasing levels of ROS caused oxidative damage to the cellular membrane. The increases in the ROS and MDA levels following exposure to the BS02 supernatant suggested that the algae had suffered from oxidative stress and were severely damaged. To avoid oxidative damage and scavenge ROS, algal cells possess a cellular defense system involving antioxidant enzymes, such as SOD and CAT (Zhang et al., 2011; Kwok et al., 2012).

Reactive oxygen species production and scavenging allow for the maintenance of homeostasis under normal metabolic conditions. However, when cells suffer from environmental stress, such as desiccation (Kumar et al., 2011), UV (Ryu et al., 2009) or high light (Solovchenko et al., 2011) or salinity (Luo and Liu, 2011), ROS metabolism becomes severely imbalanced, resulting in the accumulation of an abundance of ROS in cells, causing serious damage to the cells. Antioxidant enzymes play crucial roles in defense systems to protect cells against environmental stress. Our experiments showed that the algal cellular antioxidant enzymes (SOD and CAT) were triggered to differing degrees when cells were exposed to the BS02 supernatant (**Figure 6**). SOD and CAT activities were significantly (*p* < 0.01) enhanced compared to those of the control during the first 12 h of exposure to the 1.0 and 1.5% concentrations of the supernatant. These findings indicated that the SOD and CAT activities were activated within a short period of time. SOD activity increased gradually as the treatment time was prolonged up to 36 h. However, it decreased significantly after 36 h, implying that the BS02 supernatant stress in the algal cells was relieved. After 24 h of treatment, CAT activity began to decrease, similar to that of SOD. Although the activity of CAT decreased following the treatments with the 1.0 and 1.5% concentrations as the exposure time was prolonged, its activities at these concentrations were significantly (*p* < 0.01) higher compared with the control. These results might indicate that ROS levels were increased under BS02 supernatant stress and that the antioxidant enzymes (SOD and CAT) were activated to relieve ROS damage. In our study, the antioxidant enzymes responded actively to the BS02 supernatant effects and could relieve part of the damage caused by the ROS. However, excess ROS exceeded the functional capacities of the antioxidants and eventually led to oxidative damage.

### PIGMENT CONTENTS AND PHOTOSYNTHESIS EFFICIENCY ANALYSIS

Chlorophyll *a* plays a crucial role in algal photosynthesis and is the primary light-harvesting chromoprotein that transforms carbon dioxide into carbohydrates in algal cells (Chen, 2014). In our study, Chl *a* levels decreased significantly compared with the control after exposure to the 1.0 and 1.5% concentrations of supernatant for 24 h. In addition, as the exposure time was prolonged and the concentration was increased, this reduction was even more obvious (**Figure 7A**). These findings indicated that the pigment structures were destroyed and that the impaired pigments were rapidly degraded. Carotenoids play important roles in controlling the efficiency of light harvesting and preventing the formation of highly reactive singlet oxygen and are thus fundamental components of the photosynthetic apparatus in algae (Santabarbara et al., 2013). The carotenoid level was significantly (*p* < 0.01) decreased following 48 h of exposure to the supernatant at concentrations of 1.0 and 1.5% (**Figure 7B**). These results indicated that carotenoid synthesis was diminished and that the ability of the algal cells to resist oxidative damage was also reduced.

The variable chlorophyll fluorescence illustrated the overall photosynthetic status of the algal cells, and Fv/Fm and rETR are two important parameters used to evaluate the photosynthesis efficiency and capacity (Schreiber et al., 1995). Our results revealed that the value of Fv/Fm significantly decreased (*p* < 0.01) after exposure to the BS02 supernatant (**Figure 8A**), suggesting that it inhibited photosynthetic efficiency. Similarly, the rETR was also significantly inhibited by BS02 supernatant, implying that the photosynthetic capacity of algal cells decreased after treatment with BS02 supernatant. These results indicated that the photosynthesis systems of algal cells were attacked by the BS02 supernatant. Considering these findings and those of the pigment assays, we suggest that the BS02 supernatant may act to destroy the photosynthetic system and accelerate the degradation of pigments, thus causing the dysfunctioning of this system.

### INVOLVEMENT OF PHOTOSYNTHESIS- AND RESPIRATION-RELATED GENES IN RESPONSE TO BS02 SUPERNATANT STRESS

To reveal whether damage occurred to photosynthetic processes, the *psb*A and *psb*D genes were assessed in our study. *psb*A and *psb*D encode two core proteins, D1 and D2, which play pivotal roles in the photosynthetic reactions of PS II (Erickson et al., 1986). The BS02 supernatant greatly influenced the transcription of photosynthesis-and respiration-related genes. The expression of *psb*A increased significantly (*p* < 0.01) after 4 h of exposure to the 0.5% concentration of the supernatant in response to the reduction in chlorophyll. Within 24 h of exposure, *psb*A gene expression in all treatment groups was significantly inhibited (*p* < 0.01) compared to the control (**Figure 9A**), suggesting that the self-repair ability of the cells could not completely counteract the damage caused by the BS02 supernatant-induced stress. Within 24 h of exposure, *psb*D gene expression in all treatment groups was significantly increased (*p* < 0.01) compared to the control (**Figure 9B**), implying that the degree of repair of PS II might have increased as the concentration of BS02 supernatant increased. Although *psb*D gene expression in all treatment groups was significantly increased, the Fv/Fm values were still decreased. We speculated that the increased expression of *psb*D might be associated with the repair of PS II (Ham et al., 2010). The higher supernatant concentration induced a higher self-repair ability, and the algicidal activity was relieved by a higher activity of self-repair. Zhang et al. (2013) have reported that the transcriptional abundance of the *psb*D gene is decreased in response to BS01 supernatant, while as the concentration of the BS01 supernatant increases, *psb*D gene expression is increased. Takahashi and Badger (2011) have reported that sunlight damages PS II and causes photoinhibition, which is associated with a balance between the rate of photodamage and its repair. The photodamaged PS II is repaired through the synthesis of a new D1 protein (Murata et al., 2007); thus, the decreased expression of *psb*A may destroy the balance between the damage to PS II and its repair and the resulting inhibition of photosynthesis. The *cob* gene offers high sensitivity as a gene marker because it exists as multiple copies in many organisms (Zhang and Lin, 2005). *cob* is a cytochrome b-related gene, and *cox* is a cytochrome oxidase synthetic gene (Tobe et al., 2010). In our study, within 24 h of exposure, the expression levels of the *cob* and *cox* genes were significantly decreased (*p* < 0.05) in all treatment groups compared to the control (**Figures 9C,D**), implying that the inhibition of the respiratory system was caused by the BS02 supernatant. Yang et al. (2013) have also reported that the transcriptional abundance of the *cob* gene is significantly inhibited in response to the allelochemical hydroquinone. In summary, the transcription of photosynthesis- and respiration-related genes is influenced by the BS02 supernatant to differing degrees.

## CONCLUSION

The results of this study indicated that the algicidal bacterium strain BS02 has potential for use to control HABs. Our experiments demonstrated that changes occurred to the *A. tamarense* cells following exposure to the culture supernatant of the marine algicidal bacterium BS02. We found that the *Vibrio* sp. BS02 supernatant destroyed the morphological structure of *A. tamarense* cells, induced ROS production, inhibited Fv/Fm, and rETR, decreased pigment contents, and altered enzymatic antioxidant systems and the expression of photosynthesis- and respiration-related genes. Our study demonstrated that this supernatant affected the expression of genes involved in the photosynthesis process and might have blocked the PS II electron transport chain, leading to the production of superfluous ROS. The excess ROS inhibited algal growth and ultimately induced algal cell death.

## Conflict of Interest Statement

The authors declare that the research was conducted in the absence of any commercial or financial relationships that could be construed as a potential conflict of interest.
